# The Bell Curve Revisited: Testing Controversial Hypotheses with Molecular Genetic Data

**DOI:** 10.15195/v3.a23

**Published:** 2016-07-05

**Authors:** Dalton Conley, Benjamin Domingue

**Affiliations:** a Princeton University; b Stanford University

**Keywords:** stratification, the bell curve, dysgenics, assortative mating, educational attainment, genetics

## Abstract

In 1994, the publication of Herrnstein’s and Murray’s The Bell Curve resulted in a social science maelstrom of responses. In the present study, we argue that Herrnstein’s and Murray’s assertions were made prematurely, on their own terms, given the lack of data available to test the role of genotype in the dynamics of achievement and attainment in U.S. society. Today, however, the scientific community has access to at least one dataset that is nationally representative and has genome-wide molecular markers. We deploy those data from the Health and Retirement Study in order to test the core series of propositions offered by Herrnstein and Murray in 1994. First, we ask whether the effect of genotype is increasing in predictive power across birth cohorts in the middle twentieth century. Second, we ask whether assortative mating on relevant genotypes is increasing across the same time period. Finally, we ask whether educational genotypes are increasingly predictive of fertility (number ever born [NEB]) in tandem with the rising (negative) association of educational outcomes and NEB. The answers to these questions are mostly no; while molecular genetic markers can predict educational attainment, we find little evidence for the proposition that we are becoming increasingly genetically stratified.

More than two decades ago, the psychologist Richard Herrnstein and the policy scholar Charles Murray (hereafter H&M) penned a best-selling book that purported to explain the shifting process of economic attainment in American society (Herrnstein and Murray 1994). Their narrative essentially went as follows: earlier in the twentieth century, ascribed characteristics primarily determined who got ahead, and cognitive ability meant comparatively little. However, as institutional barriers fell over the course of generations and society became more meritocratic, achieved characteristics became more salient. So far, this account is not so different from most status attainment research or comparative mobility studies, dating back to Sibley’s (1942) classic article in the *American Sociological Review* and on through the landmark studies by Blau and Duncan (1967) and Hauser and Featherman (1976): namely, industrialization, with its market logic of efficiency, evinces a declining importance for ascriptive characteristics, as embodied by family background, and a rising salience for achieved characteristics, such as education and skills. Note Lipset and Bendix (1959:11): "Widespread social mobility has been a concomitant of industrialization and a basic characteristic of modern industrialized society. In every industrialized country, a large proportion of the population have had to find occupations considerably different from those of their parents." Or, in the words of Sibley (1942), as quoted by Blau and Duncan (1967:430): "The achieved status of a man, what he has accomplished in terms of some objective criteria, becomes more important than his ascribed status, who he is in the sense of what family he comes from." However, Herrnstein and Murray go on to suggest that an irony of the rise of meritocracy and the triumph of universalism is that cross-sectional inequality is increasingly explained by innate ability (read: genetic endowment) and therefore becomes less remediable once unfair distinctions because of unequally distributed social environments have waned in importance. That is, they argued that a genetically based caste system was coming into focus in the United States by the 1990s and was not only being reinforced by sorting in the education system and the labor market but was being solidified within the process of reproduction by an increase in assortative mating on skills and intelligence, which caused the distribution of talent to widen further with each generation.

Many critics pointed out the flaws of their provocative argument at the time, including sociologists (Fischer et al. 1996), economists (Heckman 1995), statistical geneticists (Devlin et al. 2013), and psychologists (Sternberg 1995). For example, Herrnstein and Murray (1994) were said to have overestimated the influence of genes on IQ and, in turn, of IQ on socioeconomic attainment (Devlin, Daniels, and Roeder 1997). They assumed an effect of IQ was the effect of genetic endowment, neglecting the vast literature (including from behavior genetics) showing that—at least in the U.S.—IQ is subject to large environmental influences and that even its heritability is contingent on family socioeconomic status (SES) (Turkheimer 1991; Tucker-Drob and Bates 2015). In the present study, we take three core propositions put forth by H&M and test them empirically using molecular genetic data that were not available at the time they were writing. They are as follows:

## Proposition One: The effect of genetic endowment is increasing over time with the rise of a meritocratic society

High cognitive ability as of the 1990s means, more than ever before, that the chances of success in life are good and getting better all the time, and these are *decreasingly* affected by the social environment, which by extension indicates that they must be *increasingly* affected by genes. (P. 109–110)

## Proposition Two: We are increasingly stratifying by cognitive genotypes through the process of assortative mating

Add to this phenomenon known as assortative mating. Likes attract when it comes to marriage, and intelligence is one of the most important of those likes. When this propensity to mate by IQ is combined with increasingly efficient educational and occupational stratification, assortative mating by IQ has more powerful effects on the next generation than it had on the previous one. This process too seems to be getting stronger, part of the brew creating an American class system. (P. 91–92)

## Proposition Three: Society is adversely selecting for intelligence because those with lower ability tend to have more children than those with high cognitive ability

The professional consensus is that the United States has experienced dysgenic pressures throughout either most of the century (the optimists) or all of the century (the pessimists). Women of all races and ethnic groups follow this pattern in similar fashion. There is some evidence that blacks and Latinos are experiencing even more severe dysgenic pressures than whites, which could lead to further divergence between whites and other groups in future generations. (P. 341)

H&M also make arguments about the role of genetic differences in explaining ongoing racial inequalities in U.S. society. We do not deal directly with those claims because the approach we deploy (polygenic score construction—see below) does not allow for ascertainment bias–free estimation across groups with different continental ancestries.

## Review of Polygenic Scores

Until recently, the study of human genetic variation (including the research on which Herrnstein and Murray based their conclusions) has consisted mainly of behavior genetics studies, in which twin and adoption designs were used to identify heritable, or genetic, variation in various traits (see, e.g., Plomin, Owen, and McGuffin 1994; Plomin, Haworth, and Davis 2009; Lindahl, Björklund, and Plug 2004; Sacerdote 2007). Whether or not one believes the estimates of genetic influence on social outcomes that emerge from such studies, the fact remains that they do not directly measure genotypes and thus have limited utility for stratification researchers who seek to incorporate genotype into status attainment models. Today, however, the costs of comprehensively genotyping subjects have fallen to the point where major funding bodies, including those in the social and behavioral sciences, can now begin to incorporate genetic and biological markers into major social surveys. For example, the Health and Retirement Study (HRS) has released datasets with comprehensively genotyped subjects, with others such as the National Longitudinal Survey of Adolescent to Adult Health (Add Health) and the Wisconsin Longitudinal Survey (WLS) poised to follow. Similar efforts are also underway in Europe, for example, with the Biobank Project in the United Kingdom (Ollier et al. 2005; Yuille et al. 2010) and large-scale genotyping of subjects at several European twin registries (Stene et al. 2006). These samples contain large numbers of extensively genotyped individuals and thus provide new opportunities for asking scientific questions that could not be explored until very recently. Namely, the presence of data measuring specific single nucleotide polymorphisms (SNPs) allows for what was basically impossible under the old regime of twin-based imputed heritability analysis: direct modeling of genotype as a spurious confounder or moderator of intergenerational stratification (e.g., Conley et al. 2015).

As a necessary precursor, researchers have conducted genome-wide association studies (GWASs) to measure genetic propensities for a large number of outcomes (Welter et al. 2014). A GWAS is a hypothesis-free exercise that looks for associations between a variable and millions of SNPs. In the discovery phase, a GWAS will consider regressions testing for associations between each SNP and the outcome of interest and then pool results from a consortium of data sources using meta-analysis. In what follows, we index SNPs by *j* and individuals by *i*. In the GWAS phase, each individual SNP is tested for association by running a regression of the sort:

(1) $$y_{i}=\mu_{j}+\beta_{j}x_{ij}+{Z_{i}}^{\prime }\gamma_{j}+\varepsilon_{ij}$$

where *x_ij_* is the number of reference allele individual *i* is endowed with at SNP *j* and **Z** is a vector of controls, which include age, sex, and the first four principal components (PC) of the variance–covariance matrix of the genotypic data. The PCs are included to guard against the well-known problem of population stratification: the tendency for allele frequencies to covary with unobserved environmental confounds (Price et al. 2006).

Using results from a GWAS, researchers can compile a polygenic score for a particular outcome that aggregates thousands of SNPs across the genome and weights them by the strength of their association. In essence, a polygenic score is a weighted average or composite score that takes into account information across an individual’s entire genome to measure their genetic predisposition or risk to a particular outcome. Or, a polygenic score (PGS) for individual *i* is a weighted average across the number of SNPs (*n*) of the number of reference alleles *x* (0,1, or 2) at that SNP multiplied by the score for that SNP (*β*):

(2) $${PS}_{i}=\sum_{j=1}^{n}\left(\beta_{j}x_{ij}\right)/n$$

Polygenic scores have several attractive features. First, unlike candidate genes, they are "hypothesis-free" measures—i.e., ex ante knowledge about the biological processes involved is not needed to estimate a score for a particular measure. Rather, a polygenic scores casts a wide net across an individual’s entire genome to yield a single quantitative measure of genetic risk, allowing researchers to explore how genes operate within environments where the biological mechanisms are not yet fully understood (Belsky and Israel 2014). (For other examples of PGS deployment, see, e.g., Belsky et al. [2012], Belsky et al. [2013a], Belsky et al. [2013b], Benjamin et al. [2012], Domingue et al. [2014], Domingue et al. [2015], Purcell and the International Schizophrenia Consortium [2009], Speliotes and the Genetic Investigation of Anthropometric Traits [GIANT] Consortium [2010], Yang et al. [2010].)

Recently, a GWAS was conducted for educational attainment (Rietveld et al. 2013). Using these results, a polygenic score can be constructed. When all SNPs are taken into account, this single scalar can explain between two and three percent of the variance in years of schooling. This suggests that, to the extent that it is associated with genotype, educational attainment—as we might expect—is driven by many small effects across the entire genome. This finding has further been replicated in new samples with stricter controls and the deployment of sibling fixed effects models (Rietveld et al. 2014; Conley et al. 2015; Domingue et al. 2015a). Furthermore, other polygenic scores have been shown to add predictive power over and above measured family history—at least in the health domain (Belsky et al. 2013b).

Two and a half percent is a relatively small contribution to our understanding of educational outcomes, especially when compared to the published meta-analyses of classic twin-based studies that find that genetic factors account for up to 40 percent of the variation (Branigan, McCallum, and Freese 2013). There are several important explanations for this so-called missing heritability (Manolio et al. 2009; de Los Campos et al. 2013; Dudbridge 2013), including estimation error in the coefficients from the GWAS and sample size. Specifically, these relatively low R^2^ values are largely the result of classical errors-in-variables–induced attenuation bias because of the fact that the common genotyping platforms are picking up SNPs that are in incomplete linkage (i.e., not perfectly spatially correlated) with the true causal SNPs of genetic effects. That is, X is measured with error, leading to inconsistent estimates that converge toward zero. Denser genotyping platforms and better imputation to nonmeasured SNPs, thanks to the availability of the 1000 Genomes database, to which researchers can now impute (as opposed to the older, less dense HapMap and HapMap2 platforms), may reduce this form of regression dilution to some extent. Ultimately, full genome sequencing may, of course, obviate the need for imputation and drastically reduce this form of measurement error in the Xs. A second source of imprecision is in sampling error; this reduces accuracy and is solved by increasing sample sizes (Conley 2015). Indeed, a second effort to analyze educational attainment is presently underway, with a sample size almost three times the original and with imputation to 1000 Genomes rather than HapMap2. While the PGS approach suffers from a lower explanatory power, deployment in sibling fixed effects models suggests that it is much more robust to concerns about causal inference than other approaches (see, e.g., Rietveld et al. 2014; Conley et al. 2015; Domingue et al. 2015a).

With this caveat in mind, we turn to the aims of the present study. We build on Rietveld et al. (2013, 2014) by constructing polygenic scores for HRS respondents to assess (1) whether the predictive power of genotype as measured by PGS on education (as well as income and wealth) is increasing across birth cohorts; (2) whether, as in the case of observed educational attainment (see, e.g., Schwartz and Han 2014), spouses are increasingly assorting on this genetic predictor of education across birth cohorts in the HRS; and (3) whether the PGS demonstrates a changing relationship to fertility (number of children ever born [NEB]) that mirrors the increasingly negative association between education and NEB across birth cohorts in the HRS.

## Data and Methods

SNPs in the HRS genetic database were matched to SNPs with reported results in a GWAS published by Rietveld et al. (2013) that were recalculated to exclude HRS, which had been among the original discovery samples. Because the risk allele is not always readily identifiable, we removed all ambiguous SNPs. For each of these SNPs, a loading was calculated as the number of phenotypically associated alleles multiplied by the effect size estimated in the original GWAS. SNPs with relatively large p-values will have small effects (and thus be down-weighted in creating the composite), so we do not impose a p-value threshold. Loadings were summed across the SNP set to calculate the polygenic score. The score was first residualized on the top 10 principal components (to control for population stratification) and then standardized to have a mean of 0 and a standard deviation (SD) of 1. Genetic analyses were done using the second-generation PLINK software (Chang et al. 2015).

The measure was computed based on the RAND Fat Files, version N (Chien et al. 2014), which go up to 2012, and was measured as total years of formal schooling (range 0–17). Descriptive statistics are shown in Table 1. The sample was restricted to non-Hispanic whites (N = 8,865). Respondents were born between 1919 and 1955, with a mean birth year of 1938 (SD = 9.2). Respondents received 13.2 years of education on average (SD = 2.6) and had an average of 2.6 children (SD = 1.6). Table 1 also contains descriptives for the data split by sex.

To determine whether polygenic scores are increasingly predictive over time, we consider models of the form:

(3) $${\text{Education}}_{i}=b_{0}+b_{1}{\text{PGS}}_{i}+b_{2}{\left(\text{birth}\;\text{year}-1919\right)}_{i}+b_{3}{\text{PGS}}_{i}\times {\left(\text{birth}\;\text{year}-1919\right)}_{i}+e_{i}$$

Focus is on the estimate of *b*_3_. The H&M hypothesis is that this coefficient should be positive as raw ability, in the form of genetics, becomes an increasingly stronger predictor of educational attainment over time. To test for increasing assortment through marriage, we consider models of the form:

(4) $${\text{PGS}}_{i1}=b_{0}+b_{1}{\text{PGS}}_{i2}+b_{2}{\left(\text{birth}\;\text{year}-1919\right)}_{i2}+b_{3}{\text{PGS}}_{i2}\times {\left(\text{birth}\;\text{year}-1919\right)}_{i2}+e_{i2}$$

where the left-hand side focuses on the first spouse in a spousal pair and the right-hand side focuses on the second spouse of that pair. All spousal pairs are double-entered, with the first and second spouse reversed. Huber–White standard errors are presented to account for the dependence between the paired observations. We also consider variants of Equation (3) in which the education polygenic score is replaced with realized educational attainment. Finally, in Equation (5), we probe whether there have been changes in genetic association with fertility via a modified version of Equation (3):

(5) $${\text{NEB}}_{i}=b_{0}+b_{1}{\text{PGS}}_{i}+b_{2}{\left(\text{birth}\;\text{year}-1919\right)}_{i}+b_{3}{\text{PGS}}_{i}\times {\left(\text{birth}\;\text{year}-1919\right)}_{i}+e_{i}$$

where realized educational attainment has been replaced on the left-hand side with number ever born for an individual. We also consider models in which the polygenic score on the right is replaced by realized educational attainment. Concern about standard errors robustness in Equation (1) and Equation (3) should be alleviated by the fact that we run the sexes separately in those models, in which clustering within households should not be a concern, and obtain the same findings.

## Results

### Proposition One: The effect of genetic endowment is increasing over time with the rise of a meritocratic society

To test the hypothesis that status attainment is becoming more predicted by genetic endowment, we consider simple polygenic score by birth cohort interactions (results shown in Table 2). We begin with a simplified version of Equation (3), considering only years of formal schooling and the polygenic score (see model 1 in panel A). We see that each additional standard deviation in the PGS is associated with just shy of a half-year of additional schooling among all the genotyped HRS respondents (b = 0.47, p *<* 0.001). In model 2 of panel A, we control for birth year, as those with higher scores are more likely to live longer, given the well-documented negative association between education and mortality (Cutler, Deaton, and Lleras-Muney 2006; Lleras-Muney 2005). Here, controlling for birth cohort, the effect of a one standard deviation change in PGS is 0.49 additional years of schooling, a result that is still highly significant (p *<* 0.001). In model 3 of panel A, we keep the main effect for birth year and add the interaction between the PGS and birth year. The effect for the interaction term is negative (b = –0.006, p = 0.47), suggesting that the PGS is less, rather than more, predictive of educational attainment in later birth cohorts. (Elsewhere, we demonstrate that it is highly unlikely that this effect results from selective mortality or attrition; See Conley et al. 2015a.) For an individual born in 1919, an additional standard deviation of educational genetic endowment, as measured here, results in more than half a year of schooling more on average. By the 1955 birth cohort, that effect had been reduced by one-third.

We do not believe these results are obtained because of measurement error; when we correct for measurement error, the same pattern of results remains (see Supplemental Information). Another alternative reason for our results could be that we are measuring a specific genetic profile that predicts education better in one set of birth cohorts than in another group. In other words, it could be the case that genetics do follow the pattern of increasing importance overall, but the specific measure we use shows a declining or flat effect, whereas other, unmeasured genetic profiles have ascended in importance—that we are suffering from ascertainment bias. We address this possibility in two ways. In the Supplemental Information, we show that any ascertainment bias because of age discrepancy between the discovery samples and our sample works against the pattern of results we report above. Second, we also estimated the overall SNP-based heritability of education in our data using genome-relatedness-matrix restricted maximum likelihood (GREML) models (see Yang et al. 2010 for a full explanation of this methodology) and obtained a figure of 29 percent for the group born on or before the median birth year of 1937. For the younger group born after 1937, we obtain an H^2^ of 30 percent, a difference that is not statistically significant (both heritabilities have standard errors of 0.1).

It also is possible that the functional form in Equation (3) is misspecified, or the negative linear trend we find is not robust to other specifications. However, we feel that such misspecifications are unlikely explanations of the fact that these empirical results are inconsistent the H&M hypothesis, which suggests that the observed interaction should be positive. When we split the analysis by sex in panels B and C, we see that the direction of change is the same for both sexes but appears to be greater in magnitude for men (b_3_ = –0.009 versus b = –0.003 for women). The opening of higher education to men took place earlier in the twentieth century than it did for women, thanks to the GI Bill and other policies (Stanley 2003), so this result might be expected. It would be interesting for future researchers with more recent birth cohorts—such as those sampled by the National Longitudinal Survey of Adolescent Health—to see if the period of rapid female entry into higher education and the labor market that really gained steam in the 1960s and 1970s induced a similar decline in genetic predictiveness (Goldin, Katz, and Kuziemko 2006).

In order to pinpoint where the decline in the genetic effect of education was occurring in the distribution, we conducted analysis in which we estimated linear probability models for educational transitions (see Mare 1980). These results are shown in Table 3. We focus on at least finishing high school (≥12 years of attainment), at least some college (>12 years of attainment), finishing college or more (≥16 years of attainment), or more than college (>16 years of attainment). Note that for each educational stage, our analyses tend to focus on those who have completed the step below (i.e., we look at those who attend at least some college amongst only those who graduated high school). The means for these outcomes in the conditional analyses are also shown in the table.

When we examine the strength of the educational PGS as a predictor across educational transitions (i.e., a main effect constrained to be constant across birth cohorts), we find that it increases as we move from high school completion to the transition to and completion of college (Table 3, panel A). However, when we model the transition from college to graduate school, it once again declines. In panel B of Table 3, we examine the interaction between the PGS and birth year. Here, we find that it is at the lower end of the distribution (high school graduation) where the effect of the PGS is declining—a finding that is in line with maximally maintained inequality theory (see Discussion section). Indeed, at the highest educational transition—from college completion to graduate school—we find that the effect of genetics is increasing in younger birth cohorts. (We also tested these models using a PGS from the same consortium [Rietveld et al. 2013] specifically meant to predict college completion and got the same result; see Supplemental Information.)

### Proposition Two: We are increasingly stratifying by cognitive genotypes through the process of assortative mating

We assess proposition two via estimates of variants of Equation (4) (see Table 4). We begin with a simplified version (model 1) in which we regress one spouse’s education level on the other’s, controlling for birth year (estimating Huber–White robust standard errors to account for the double entry of spouses on each side of the equation). In model 2, we add the interaction between spouse’s birth year and education. The interaction effect is significant and positive (b = 0.005, p = 0.01), suggesting that more recent cohorts assort on education more than older cohorts—a finding that reproduces a well-documented dynamic (see, e.g., Schwartz 2014 for a review) and which, absent molecular genetic data, could be seen as evidence in support of the H&M proposition. However, in model 3 of Table 4, we estimate the same model, but instead of realized education level, we substitute education PGS and find that spouses do also assort on their education genotype (b = 0.13, p *<* 0.001), a finding consistent with earlier work showing general spousal assortment on genotypes (Domingue et al. 2014a; Guo et al. 2014). But in model 4, in which we add the interaction effect between spouse’s PGS and birth year, we find that—directly in contradiction to both the trend with respect to phenotypic assortment as well as to the H&M hypothesis—the similarity between spousal educational PGS declines in more recent cohorts (b = –0.003), though not significantly so when we consider the Huber–White robust standard errors. For education level, an additional year of schooling would translate to 0.41 additional years of schooling for one’s spouse on average for those born in 1919 and only 0.61 years of additional schooling for one’s spouse if born in 1955. Meanwhile, for those born in 1919, an additional standard deviation of educational genetic endowment would lead to a marriage to someone with an additional 0.19 standard deviations on the educational PGS; by 1955, that effect would have declined to 0.08 standard deviations. While we are underpowered to generate a precise enough estimate of the trend that excludes zero, we again emphasize that the valence of our empirical evidence is contrary to the claims of H&M.

### Proposition Three: Society is adversely selecting for intelligence because those with lower ability tend to have more children than those with high cognitive ability

Since the demographic transition, it has been the case that those with more education—particularly when it comes to women—have lower total fertility rates than those with less education (Balbo, Billari, and Mills 2013; Rindfuss, Morgan, and Offutt 1996). These fertility differences could give rise to dysgenic dynamics, whereby the genetic distribution of the population is increasingly skewed toward those with less educational potential. However, this would only hold true if the differential pattern of fertility held at the genotypic level as well as the phenotypic one. In Table 5, we show that not only do those with less formal schooling have fewer children (model 1, panel A) but also that this relationship is stronger for women than for men (contrasting model 1 in panels B and C). Further, the decline in fertility for more educated individuals has become more pronounced in more recent birth cohorts (for both sexes), as evidenced by a negative interaction effect between birth year and education level in model 2 of panels A (combined), B (men), and C (women). In the pooled sample in 1919, an individual with 10 years of schooling would be predicted to have had 3.6 children, while if that person had 15 years of schooling, he or she would be predicted to have had 3.5 children. However, by the 1955 birth cohort, that same individual with 10 years of schooling would have borne 2.3 children on average, while the respondent with 15 years was predicted to have had 1.6 children.

When we turn to genotype, we again find that the genetic trend is contrary to the trend observed working with realized educational attainment. There does appear to be a modest negative effect of educational PGS on number of children ever born (model 3 of panel A; b = –0.067, p *<* 0.001), such that an additional standard deviation in educational PGS results in 1/15*^th^* fewer children on average. More importantly, however, model 4 of panel A shows that the interaction between birth year and PGS—the direct test of whether such dynamics are shifting over time as H&M suggest—is insignificant. Therefore, as a society, we do not seem to be experiencing increasing dysgenic dynamics, despite the fears of H&M, among others (Lynn 2011). When we break out this analysis by sex in panels B and C of Table 5, we find that dynamics are also insignificant for each group.

## Discussion

*The Bell Curve* argued that we have achieved the societal situation in which the result of meritocracy and assortative mating was a system of class stratification based on innate (i.e., genetic) endowment (Herrnstein and Murray 1994), as first satirically proposed by Michael Young back in 1958 when the term "meritocracy" was coined (Young 1958). If this were true, social policy to promote equal opportunity would be counterproductive—at least on efficiency grounds—because each individual will have reached the level of social status best suited to the individual’s native abilities. Meanwhile, by selectively breeding with others of similar genetic stock, parents would reinforce their offspring’s advantages or disadvantages. Such a situation would call into question the notion that intergenerational correlations in SES variables—such as income, occupation, or education—reflect a lack of meritocratic openness in a given society.

Herrnstein and Murray made their assertions in 1994, before the human molecular genetics revolution took place. Whatever one believed about their assertions or the politics thereof, they were largely untestable at the time. They based their claims on analysis of IQ, which is problematic because IQ has both environmental and genetic bases, so any trend in its effects could be attributable to the environmentally influenced portion or the genetically determined one. Further, they analyzed the National Longitudinal Survey of Youth 1979 (NLSY79)—a survey of men and women born in the years 1957–1964. This is hardly a wide enough swath of birth cohorts (particularly because everyone was born postwar) to test their grand theory. By contrast, we test their hypotheses using molecular data across a much wider (and frankly, more appropriate) birth cohort distribution.

With this in mind, the present article examined three empirical phenomena that flow from this argument and fail to find much corroborating evidence: (1) the correlation between education genotype and years of schooling is not increasing across birth cohorts in the twentieth century (only for the transition from college to graduate school does genetics appear to have increased in importance); (2) assortative mating on the underlying genetic architecture for educational attainment is flat, not increasing as phenotypic educational assortment seems to be; and (3), there is no change in the relative fertility rate by education genotype across birth cohorts—i.e. we do not appear to have entered a period of "dysgenics." We do not believe our results are driven by ascertainment bias or random measurement error (see Supplemental Information) or by selective mortality bias (see Conley et al. 2015a). Moreover, we find that patterns observed with respect to the realized outcome (i.e. educational attainment) are frequently opposite the patterns observed for the polygenic score associated with this measure.

Our analysis also speaks to debates over the impact of family background within the education system. Mare (1980) suggests that the further up the educational "ladder" a given transition lies, the less family background should matter because of selection gradients at each stage. While educational PGS is part of one’s patrimony, it demonstrates significant variation within families and, indeed, is arguably a measure of the universalistic, skill-based endowment on which the selection gradient of universalism should operate. We found that across all birth cohorts, the effect of the educational PGS is strongest for college attainment and weaker for high school completion and graduate transitions—thus demonstrating an inverse U-shaped pattern that does not conform to the selection gradient theory.^1^ Deploying this educational stage analysis, we also tested a genetic version of maximally maintained inequality theory (MMI) (see Raftery and Hout 1993). A prediction of MMI theory is that as a given educational stage (such as secondary school) approaches saturation (i.e., is universally accessible), class background should matter less for that stage. To examine this proposition while substituting genetic profile for family background, we analyzed the interaction effect between educational PGS and birth cohort by stage. As secondary schooling became universally accessible, we should see the decline of the PGS effect for high school graduation over time, but because postsecondary schooling did not expand to the same inequality-dampening degree, for transitions within the postsecondary system, we would expect no decline in the impact of the PGS in younger birth cohorts. Here, in line with theories of maximally maintained inequality, we find that the decline in the importance of genetic profile is seen in the lower half of the educational distribution (primarily high school completion and transition to postsecondary schooling). Indeed, we find that for transition from a completed college degree to graduate education, the trend of the effect of the PGS is positive over time.

In conclusion, though we are less than two decades away from the 2033 date at which Young had predicted the final "revolt" against an entrenched meritocratic system (in the United Kingdom), it seems—if the present results are to be believed—we are still a long way away from that dystopia he painted over a half century ago.

## Supplementary Material

Supplemental Material

## Figures and Tables

**Table 1:** Descriptive Statistics

	Minimum	Maximum	Mean	Standard Deviation
	All (N = 8,865)			
Birth Year	1919	1955	1938	9
Education (Years)	0	17	13.18	2.57
NEB	0	14	2.59	1.62
	Males (N = 3,809)			
Birth Year	1919	1955	1937	9
Education (Years)	0	17	13.40	2.78
NEB	0	14	2.55	1.61
	Females (N = 5,056)			
Birth Year	1919	1955	1938	9.35
Education (Years)	0	17	13.01	2.39
NEB	0	13	2.62	1.63

**Table 2:** Effect of Educational Polygenic Score (PGS) on Educational Attainment across Birth Cohorts in the HRS

	(1)	(2)	(3)
Panel A: Full Sample			
(Intercept)	13.2†	12.3†	12.3†
	(0.027)	(0.060)	(0.060)
PGS	0.469†	0.485†	0.594†
	(0.027)	(0.027)	(0.061)
Birth Year		0.046†	0.046†
		(0.003)	(0.003)
Birth Year X PGS			−0.006*
			(0.003)
N	8,851	8,851	8,851
*R*^2^	0.033	0.06	0.061
Panel B: Males Only			
(Intercept)	13.4†	12.5†	12.5†
	(0.044)	(0.101)	(0.101)
PGS	0.480†	0.495†	0.662†
	(0.044)	(0.043)	(0.100)
Birth Year		0.046†	0.047†
		(0.005)	(0.005)
Birth Year X PGS			−0.009*
			(0.005)
N	3,801	3,801	3,801
*R*^2^	0.031	0.053	0.054
Panel C: Females Only			
(Intercept)	13.0†	12.1†	12.1†
	(0.033)	(0.073)	(0.073)
PGS	0.456†	0.474†	0.538†
	(0.034)	(0.033)	(0.075)
Birth Year		0.047†	0.047†
		(0.004)	(0.004)
Birth Year X PGS			−0.003
			(0.004)
N	5,050	5,050	5,050
*R*^2^	0.035	0.069	0.069

* p < 0.05;

† p < 0.01

**Table 3:** Effect of Educational Polygenic Score on Educational Attainment across Birth Cohorts in the HRS, by Educational Stage

Outcome	Education (Years)	Finished High School (or more)	Some College (or more)	Finished College (or more)	Finished College (or more)	More Than College
For those who had	Any	Any	Finished High School	Finished High School	Some College	Finished College
Mean Outcome	13.2	0.85	0.58	0.31	0.53	0.53
Panel A: Eqn 3 without interaction						
(Intercept)	12.30†	0.75†	0.46†	0.23*	0.50†	0.51*
	(0.060)	(0.008)	(0.013)	(0.012)	(0.018)	(0.025)
PGS	0.485†	0.036†	0.076†	0.079†	0.066†	0.024†
	(0.027)	(0.003)	(0.006)	(0.005)	(0.007)	(0.01)
Birth Year	0.046†	0.0054†	0.006†	0.004†	0.001	0.000
	(0.003)	(4e-04)	(0.001)	(0.001)	(0.001)	(0.001)
N	8,851	8,851	7,537	7,537	4,334	2,304
*R*^2^	0.06	0.029	0.034	0.034	0.018	0.0025
Panel B: Eqn 3						
(Intercept)	12.30†	0.75†	0.46†	0.23*	0.50†	0.53*
	(0.06)	(0.008)	(0.013)	(0.012)	(0.018)	(0.026)
PGS	0.590†	0.066†	0.093†	0.073†	0.054†	−0.029
	(0.061)	(0.008)	(0.013)	(0.012)	(0.017)	(0.024)
Birth Year	0.046†	0.005†	0.006†	0.004†	0.001	−0.000
	(0.003)	(4e-04)	(0.001)	(0.001)	(0.001)	(0.001)
Birth Year X PGS	−0.006*	−0.002†	−0.001	0.000	0.001	0.003*
	(0.003)	(4e-04)	(0.001)	(0.001)	(0.001)	(0.001)
N	8,851	8,851	7,537	7,537	4,334	2,304
*R*^2^	0.061	0.03	0.034	0.034	0.018	0.0051

* p < 0.05;

† p < 0.01

**Table 4:** Assortative Mating by Years of Schooling and Educational PGS across Birth Cohorts in the HRS

	Education (Years)		PGS	
	(1)	(2)	(3)	(4)
(Intercept)	6.193†	7.538†	0.075*	0.070*
	(0.178)	(0.406)	(0.036)	(0.036)
Education	0.514†	0.414†		
	(0.013)	(0.030)		
Birth Year	0.016†	−0.058*	−0.003	−0.003
	(0.004)	(0.020)	(0.002)	(0.002)
Birth Year X Education		0.005†		
		(0.001)		
PGS			0.131†	0.193†
			(0.014)	(0.036)
Birth Year X PGS				−0.003
				(0.002)
N	4,676	4,676	4,676	4,676
*R*^2^	0.27	0.27	0.018	0.019

NOTE: Huber–White standard errors are in parentheses and are heteroskedasticity-consistent.

* p < 0.05;

† p < 0.01

**Table 5:** Effect of Years of Schooling and Educational PGS on Number of Children Ever Born across Birth Cohorts in the HRS

	(1)	(2)	(3)	(4)
Panel A: Full Sample				
(Intercept)	4.63†	3.74†	3.62†	3.62†
	(0.096)	(0.233)	(0.047)	(0.047)
Education	−0.082†	−0.015		
	(0.007)	(0.017)		
Birth Year	−0.047†	−0.000	−0.051†	−0.051†
	(0.002)	(0.011)	(0.002)	(0.002)
Birth Year X Education		−0.004†		
		(0.001)		
PGS			−0.067†	−0.13†
			(0.017)	(0.047)
Birth Year X PGS				0.003
				(0.002)
N	7,980	7,980	7,994	7,994
*R*^2^	0.083	0.085	0.068	0.068
Panel B: Males Only				
(Intercept)	4.08†	3.75†	3.56†	3.56†
	(0.136)	(0.313)	(0.067)	(0.067)
Education	−0.042†	−0.017		
	(0.010)	(0.023)		
Birth Year	−0.050†	−0.031*	−0.052†	−0.052†
	(0.003)	(0.016)	(0.003)	(0.003)
Birth Year X Education		−0.001		
		(0.001)		
PGS			−0.065*	−0.21†
			(0.026)	(0.066)
Birth Year X PGS				0.008*
				(0.003)
N	3,547	3,547	3,555	3,555
*R*^2^	0.075	0.075	0.071	0.073
Panel C: Females Only				
(Intercept)	5.18†	4.06†	3.70†	3.70†
	(0.136)	(0.355)	(0.065)	(0.065)
Education	−0.124†	−0.037		
	(0.010)	(0.027)		
Birth Year	−0.046†	0.010	−0.052†	−0.052†
	(0.003)	(0.016)	(0.003)	(0.003)
Birth Year X Education		−0.004†		
		(0.001)		
PGS			−0.067†	−0.047
			(0.024)	(0.066)
Birth Year X PGS				−0.001
				(0.003)
N	4,433	4,433	4,439	4,439
*R*^2^	0.097	0.100	0.068	0.068

* p < 0.05;

† p < 0.01

## Footnotes

1 This pattern is (perhaps coincidentally) inversely parallel to the pattern found by Torche (2011) with respect to the impact of family background on occupational outcomes: at low education levels, intergenerational social reproduction is high, for college graduates it is lowest, and for those with graduate education, the impact of family background on occupational outcomes strengthens again.
