# Supplementary material for: The Bell Curve Revisited: Testing Controversial Hypotheses with Molecular Genetic Data
Source: Sociol Sci. Author manuscript; Available in PMC 2017 Nov 9. (PMC5679002; doi:10.15195/v3.a23)
Supplement: Supplemental Material [file NIHMS883653-supplement-Supplemental_Material.pdf]

Supplement to:

Conley, Dalton, and Benjamin Domingue. 2016. "The Bell Curve Revisited: Testing Controversial Hypotheses with Molecular Genetic Data" Sociological Science 3: 520-539.

The Bell Curve Revisited:

Testing Controversial Hypotheses with Molecular Genetic Data

Supplemental Information

Dalton Conley[1] & Benjamin Domingue[2]

Contents:

Supplementary Note................................Page 2

Supplementary Tables.............................Page 5

Supplementary References.....................Page 12

[1] Department of Sociology, Princeton University, Princeton, NJ 08644
[2] Graduate School of Education, Stanford University, Stanford CA94305

Supplementary Note

A. Discovery Sample Ascertainment Bias

It could theoretically be possible that any changes across cohorts in the predictive accuracy of polygenic scores are not the result of changes in the overall genetic component of the phenotypic variation (i.e. increasing or decreasing genetic effects or assortment) but rather the fact that those particular alleles that are associated with either phenotype or spousal genotype in one cohort are not predictive in another cohort (while alternative scores would be equally assortative or predictive). That is, it could be that any changes across birth cohorts merely reflect ascertainment bias due to the birth cohort distribution of the discovery samples. Namely, even if genetic factors as a whole are similarly important across birth cohorts, if *different* genetic factors matter for different birth cohorts, then a PGS constructed with weights from younger birth cohorts may have worse predictive power in older birth cohorts. Specifically, if the discovery analysis was done on younger birth cohorts, it would come as no surprise that the score predicts better for the younger group in the HRS (or vice versa).

To find out whether this was driving the dynamics report here, we went back to the original studies that formed the bases of the meta-analyses of the consortia GWAS on which our polygenic scores are based. Information about the birth date distribution was available for all of studies for all cohorts. From these, we computed a weighted average of the birth year for the discovery sample of that particular polygenic score. These birth cohort means are reported in Table S1, below. The mean summarized from that table are as follows: 1947. Thus, the mean year of birth was more recent than the median in our sample (1938). Thus, any ascertainment bias would work in the direction of finding greater predictive power in later cohorts. That is, since our PGS is estimated on younger cohorts on average, then this source of bias would cause it to predict education better for the younger cohorts in the HRS. Thus, this source of bias is also unlikely to drive the pattern we show above.

B. Random Measurement Error

A second potential concern with respect to estimating the effects of polygenic scores is the fact that polygenic scores are measured with error that may lead to attenuation bias. We used SIMEX (Stefanski & Cook, 1995) to correct for potential attenuation bias. This worked as follows:

1. We first estimate additive heritabilities via GCTA (Yang et al. 2011) for the four outcomes. Denote these h^2_{GCTA}.
2. We then assume that, for each outcome

$$y = g_t + e_1$$

where g_t is the unobserved true polygenic score and is related to the heritability via the following expression:

$$h^2_{GCTA} = \frac{\mathrm{Var}(g_t)}{\mathrm{Var}(y)}.$$

We then assume that

$$g_o = g_t + e_2$$

whereg_o is the observed polygenic score. Usage of SIMEX requires an estimate for $\mathrm{Var}(e_2)$. We obtain that by first noting that

$$\mathrm{Var}(g_t) = h^2_{GCTA}\mathrm{Var}(y)$$

And then using

$$\mathrm{Var}(e_2) = \mathrm{Var}(g_o) - h^2_{GCTA}\mathrm{Var}(y)$$

by standard assumptions on e_2.
3. This estimate for $\mathrm{Var}(e_2)$ is then used in SIMEX to simulate predictors

$$g_S \sim \mathrm{Normal}[g_o, (1 + \lambda)\mathrm{Var}(e_2)].$$

For different values of λ (typically over a grid between 0 and 2), a trend in the estimates of the relevant covariates is established which is then extrapolated back to the case where $\lambda = -1$. Under certain assumptions (e.g., additive measurement error) that are reasonable in the context of polygenic scores, this is the unbiased estimator.

Results from this analysis are shown in Tables S2. In all cases, the main effect of polygenic score and the interaction of polygenic score and birth year increase in

magnitude after correction via SIMEX. This is accompanied by increases in the associated SE so there is frequently not a dramatic change in the associated p-value. These analyses suggest that measurement error in the polygenic scores is unlikely to underestimate the true genetic dynamic but, as expected, would not lead to sign reversals.

C. Results based on polygenic score for college graduation.

We constructed a second polygenic score based on GWAS results for college completion from the same GWAS (Rietveld et al 2013). This was then used to conduct analyses similar to those from Table 3 Panel B of the main text. The findings were largely consistent with those from the main text.

Table S1: Birth cohorts for discovery samples--Social Science Genetics Association Consortium (SSGAC), Education

PERCENT TOTAL SAMPLE, BIRTHYEAR ASCERTAINED: 100%

WEIGHTED AVERAGE BIRTHYEAR: 1947.271

Study	Year(s)	Ages [mean,sd]	Birth Cohort	N
Age, Gene/Environment Susceptibility-Reykjavik Study (AGES)	Baseline 2002	66-95 [76.41, 5.45]	1908-1936<br>Mean: 1926	3,212
Avon Longitudinal Study of Parents and Children (ALSPAC)		15-44 [28.69, 4.66]	1948-1977<br>Mean: 1962.95	6,919
Austrian Stroke Prevention Study (ASPS)		46-85 [65.51, 7.98]	1909-1949<br>Mean: 1931.97	848
Baltimore Longitudinal Study of Aging (BLSA)		30-101 [71.63, 15.55]	1902-1977<br>Mean: 1933.9	821
Cancer Hormone Replacement Epidemiology in Sweden (CAHRES)		66-92 [79, 6.2]	1919-1944<br>Mean: 1931	1,497
Cancer Prostate Sweden (CAPS)		49-81 [68, 7.5]	1921-1954<br>Mean: 1933 (Cases); 1935 (Controls)	459

Cleveland Clinic Foundation (CCF)		30-84 [59.39, 9.82]	1923-1978<br>Mean: 1947	485
CohorteLausannoise (CoLaus)	2003	34-75 [53.43, 10.75]	1928-1970<br>Mean: 1951	5410
Croatia Korcula (CRKOR)		30-98	1909-1979<br>Mean: 1949 (Korcula); 1955.9 (Split); 1944.8 (Vis)	2,124
Estonian Genome Center, University of Tartu (EGCUT)	2001 baseline (to 2007, rolling? not defined)	30-103	1905-1979<br>Mean: 1949	1,537
Erasmus Rucphen Family-EUROSPAN (ERF)	Baseline 2002 (-2005)	30-89 [51.77, 12.29]	1914-1974<br>Mean: 1951	2,380
Finnish FINRISK (FINRISK)		30-74 [46.28, 11.40]	1923-1977<br>Mean: 1946	1,823
Finnish Twin Cohort (FTC)		41-67 [54.88, 4.48]	1937-1961<br>Mean: 1948	729
Genetic Association Information Network Schizophrenia Controls (GAIN)		30-90 [55.48, 14.23]	1916-1976<br>Mean: 1950	1,164

Genetic Epidemiology Network of Arteriopathy (GENOA)	24-89 [55.33, 10.82]	1908-1974<br>Mean: 1942	1,439
Health ABC (HABC)	69-80 [73.77, 2.84]	1917-1928<br>Mean: 1923	1,659
Helsinki Birth Cohort Study (HBCS)	56-69 [61.47, 2.92]	1934-1944<br>Mean: 1940.85	1,717
Invecchiare in Chianti (InCHIANTI)	30-102 [69.95, 13.29]	1896-1970<br>Mean: 1928	1,164
KooperativeGes undheitsforschu ng in der Region Augsburg (KORA3)	30-69 [53.28, 9.20]	1925-1964<br>Mean: 1940	1,595
KooperativeGes undheitsforschu ng in der Region Augsburg (KORA4)	31-74 [53.93, 8.82]	1926-1969<br>Mean: 1946	1,809
Lifelines Cohort Studies (LIFELINE)	30-89 [48.57, 10.31]	1920-1980<br>Mean: 1959.97	7,493
Lothian Birth Cohort 1921 (LBC1921)	77-80	1921 Only	515
Lothian Birth Cohort 1936 (LBC1936)	67-71	1936 Only	1,003

Mother and Child Cohort of NIPH (MoBa)	20-34	1966-1976<br>Mean: 1971	759
Netherlands Study of Depression and Anxiety (NESDA)	30-65 [46.69, 9.35]	1939-1976<br>Mean: 1959	1,517
Northern Finland Birth Cohort 1966 (NFBC1966)	31	1966 Only	5,371
Non-Genetic Association Information Network Schizophrenia (nonGAIN)	30-90 [53.02, 13.88]	1916-1976<br>Mean: 1953	1,109
Netherlands Twin Register (NTR)	30-91 [51.39, 12.49]	1917-1980<br>Mean: 1954	2,650
Queensland Institute of Medical Research (QIMR)	30-101 [44.95, 10.09]	1900-1975<br>Mean: 1951	7,985
Rotterdam Study Baseline (RSI)	55-99 [69.18, 8.95]	1893-1938<br>Mean: 1922	5,806
Rotterdam Study Extension (RSII)	55-95 [64.97, 8.15]	1906-1944<br>Mean: 1935	1,641
Rotterdam Study Young (RSIII)	45-97 [56.11, 5.84]	1910-1960<br>Mean: 1950	2,014

Rush University Medical Center-Memory and Aging Project (RUSH-MAP)	55-101 [81.10, 6.66]	1901-1948<br>Mean: 1921	888
Rush University Medical Center-Religious Orders Study (RUSH-ROS)	60-102 [75.70, 7.35]	1896-1946<br>Mean: 1921	810
Study of Addiction: Genetics and Environment (SAGE)	30-65 [38.70, 5.68]	1938-1975<br>Mean: 1965	1,321
SardiNIA Study of Aging (SardiNIA)	30-101 [52.81, 14.25]	1900-1980<br>Mean: 1954	3,639
Study of Health in Pomerania (SHIP)	30-81 [53.27, 14.08]	1918-1971<br>Mean: 1945	3,556
Swedish Twin Register (STR)	47-89 [63.82, 8.74]	1916-1958<br>Mean: 1941	9,553
UK Adult Twin Registry (TwinsUK)	30-80 [51.03, 10.72]	1919-1978<br>Mean: 1949	2,619
Cardiovascular Risk in Young Finns Study (YFS)	30-45 [37.72, 5.01]	1962-1977<br>Mean: 1969	2,029

Table S2: SIMEX Results. Education is standardized so coefficient estimates will not match those from main text.

		Original Results				SIMEX			
		Est	SE	t	P-value	Est	SE	t	P-value
		Table 2							
Column 1	(Intercept)	-4.22E-05	0.0105	-0.00403	9.97E-01	-0.00022	0.0104	-0.0207	9.83E-01
	PGS	1.82E-01	0.0105	17.42976	6.38E-67	0.302786	0.0157	19.2629	4.95E-81
Column 2	(Intercept)	-0.336	0.02336	-14.4	2.28E-46	-0.3461	0.02331	-14.8	2.95E-49
	PGS	0.189	0.01031	18.3	2.22E-73	0.314	0.01524	20.6	3.60E-92
	Birth Year	0.018	0.00112	16	5.62E-57	0.0185	0.00112	16.5	1.58E-60
Column 3	(Intercept)	-0.338	0.02338	-14.46	7.52E-47	-0.34831	0.02352	-14.8	5.10E-49
	PGS	0.23083	0.0236	9.78	1.76E-22	0.38363	0.03455	11.1	1.84E-28
	Birth Year	0.01804	0.00112	16.08	2.28E-57	0.01859	0.00113	16.5	3.60E-60
	Birth Year X PGS	-0.00225	0.00113	-1.99	4.69E-02	-0.00385	0.00167	-2.3	2.14E-02
		Table 3							
Column 3	(Intercept)	0.07496	0.036	2.08	3.74E-02	0.06626	0.03598	1.84	6.56E-02
	PGS	0.13133	0.0145	9.06	1.94E-19	0.22057	0.02021	10.91	2.15E-27
	Birth Year	-0.00315	0.0017	-1.86	6.30E-02	-0.00281	0.00169	-1.66	9.76E-02
Column 4	(Intercept)	0.07017	0.03609	1.94	5.19E-02	0.0601	0.03619	1.66	9.69E-02
	PGS	0.19353	0.03653	5.3	1.23E-07	0.32876	0.05295	6.21	5.81E-10
	Birth Year	-0.00295	0.0017	-1.73	8.29E-02	-0.00252	0.0017	-1.48	1.38E-01
	Birth Year X PGS	-0.00317	0.00171	-1.86	6.36E-02	-0.00572	0.00252	-2.27	2.32E-02
		Table 4							
Column 3	(Intercept)	3.6239	0.04678	77.47	0.00E+00	3.6255	0.04676	77.53	0.00E+00
	PGS	-0.0669	0.01747	-3.83	1.29E-04	-0.1132	0.02555	-4.43	9.46E-06
	Birth Year	-0.0512	0.00214	-23.94	2.40E-122	-0.0513	0.00214	-23.99	7.66E-123
Column 4	(Intercept)	3.62395	0.04677	77.48	0.00E+00	3.62542	0.04678	77.5	0.00E+00
	PGS	-0.12708	0.04669	-2.72	6.51E-03	-0.21473	0.06894	-3.11	1.85E-03
	Birth Year	-0.0512	0.00214	-23.93	2.84E-122	-0.05128	0.00214	-23.97	1.27E-122
	Birth Year X PGS	0.00298	0.00214	1.39	1.65E-01	0.00534	0.00307	1.74	8.18E-02

Table S3: Effect of College polygenic score on education attainment across birth cohorts in the HRS by educational stage

Outcome	Education (Years)	Finished High School (or more)	Some College (or more)	Finished College (or more)	Finished College (or more)	More than college
For those who had	Any	Any	Finished High School	Finished High School	Some College	Finished College
Mean Outcome	13.2	0.85	0.58	0.31	0.53	0.53
(Intercept)	12***	0.75***	0.46***	0.23***	0.5***	0.52***
	(0.06)	(0.0084)	(0.013)	(0.012)	(0.018)	(0.026)
PGS	0.52***	0.061***	0.067***	0.057***	0.047**	-0.012
	(0.06)	(0.0084)	(0.013)	(0.012)	(0.017)	(0.023)
Birth Year	0.046***	0.0054***	0.0056***	0.0035***	0.00085	-0.00028
	(0.0029)	(4e-04)	(0.00061)	(0.00057)	(0.00081)	(0.0012)
Birth Year X PGS	-0.0012	-0.0011**	0.00025	0.001+	0.00087	0.0023*
	(0.0029)	(4e-04)	(6e-04)	(0.00056)	(0.00077)	(0.001)
N	8851	8851	7537	7537	4334	2304
R2	0.062	0.032	0.031	0.033	0.018	0.0064

References

Rietveld, C. A., Medland, S. E., Derringer, J., Yang, J., Esko, T., Martin, N. W., ... & Albrecht, E. (2013). GWAS of 126,559 individuals identifies genetic variants associated with educational attainment. science, 340(6139), 1467-1471.

Stefanski, L. A., & Cook, J. R. (1995). Simulation-extrapolation: the measurement error jackknife. Journal of the American Statistical Association, 90(432), 1247-1256.

Yang, J., Lee, S. H., Goddard, M. E., & Visscher, P. M. (2011). GCTA: a tool for genome-wide complex trait analysis. The American Journal of Human Genetics, 88(1), 76-82.
